# Designing *P. aeruginosa* synthetic phages with reduced genomes

**DOI:** 10.1038/s41598-021-81580-2

**Published:** 2021-01-25

**Authors:** Diana P. Pires, Rodrigo Monteiro, Dalila Mil-Homens, Arsénio Fialho, Timothy K. Lu, Joana Azeredo

**Affiliations:** 1 CEB - Centre of Biological Engineering, Universidade Do Minho, Campus de Gualtar, Braga, Portugal; 2 Institute for Bioengineering and Biosciences (iBB), Instituto Superior Técnico, Lisboa, Portugal; 3 Department of Bioengineering, Instituto Superior Técnico, Universidade de Lisboa, Lisboa, Portugal; 4 Department of Electrical Engineering and Computer Science and Department of Biological Engineering, Synthetic Biology Center, Massachusetts Institute of Technology, 77 Massachusetts Avenue, Cambridge, MA 02139 USA

**Keywords:** Bacteriology, Bacteriophages, Antimicrobials, Synthetic biology

## Abstract

In the era where antibiotic resistance is considered one of the major worldwide concerns, bacteriophages have emerged as a promising therapeutic approach to deal with this problem. Genetically engineered bacteriophages can enable enhanced anti-bacterial functionalities, but require cloning additional genes into the phage genomes, which might be challenging due to the DNA encapsulation capacity of a phage. To tackle this issue, we designed and assembled for the first time synthetic phages with smaller genomes by knocking out up to 48% of the genes encoding hypothetical proteins from the genome of the newly isolated *Pseudomonas aeruginosa* phage vB_PaeP_PE3. The antibacterial efficacy of the wild-type and the synthetic phages was assessed in vitro as well as in vivo using a *Galleria mellonella* infection model. Overall, both in vitro and in vivo studies revealed that the knock-outs made in phage genome do not impair the antibacterial properties of the synthetic phages, indicating that this could be a good strategy to clear space from phage genomes in order to enable the introduction of other genes of interest that can potentiate the future treatment of *P. aeruginosa* infections.

## Introduction

Antibiotic resistance is rising to high levels all around the world, being currently considered as one of the major threats to human health^1^. This fact has triggered an increasing interest on alternative therapeutic approaches including phage therapy^2^. Bacteriophages have been broadly explored for a wide range of applications but despite all the efforts that have been made to bring phages into the clinical settings, phage therapy still faces some challenges such as limited host ranges, bacterial resistance to phages, side effects of bacterial lysis, as well as safety, stability and delivery issues^3–5^. Phage engineering holds a great potential to generate phage variants with improved antibacterial properties, which might contribute to the enhancement of phage therapy. This is usually achieved by cloning additional genes into the phage genomes^6^, which might be difficult without removing unnecessary phage-encoded genes. Considering that a very large proportion of phage genomes encode for hypothetical proteins with unknown functions^7^, a possible approach to get some space for genome engineering would be the precise removal of those genes from viral genomes. In addition, for safety purposes, it has been advised that phages carrying genes encoding toxins, virulence factors or antibiotic resistance should be avoided from therapeutic applications^8^. Therefore, it would be better for therapeutic purposes to have phages carrying a minimum number of genes with unknown functions, as we still don’t know if those genes can be potentially hazardous. Although transcriptomic studies on phage-bacteria interaction are bringing important clues to understand the role of the hypothetical proteins encoded on phage genomes^9–11^, their functional analysis is still challenging and can be time consuming. Here, we envisioned that synthetic phages carrying a minimal number of genes essential for their replication and host killing, would be more easily accepted for therapy and a minimal genome concept would also facilitate the integration of extra functions into the phage genome to improve its performance.

The recent advances in genetic engineering and sequencing technologies have led to a fast development of phage-engineering tools that can now be applied in a high throughput fashion to design and build synthetic phages with desirable features^5,6^. Taking advantage of the yeast-based phage-engineering platform^12^, we aimed to create synthetic phages with reduced genomes, minimizing the number of genes encoding hypothetical proteins. Since *P. aeruginosa* is currently considered by the World Health Organization as one of the top priority bacterial pathogens urgently requiring new treatments^13^, in this study we isolated a new *P. aeruginosa* phage and used it as a model to design our synthetic phages. From our knowledge, this is the first study focused on minimizing phage genomes and understanding its impact on phages’ performance.

## Results

### Characterization of the newly isolated *P. aeruginosa* phage vB_PaeP_PE3

**Figure 1:**
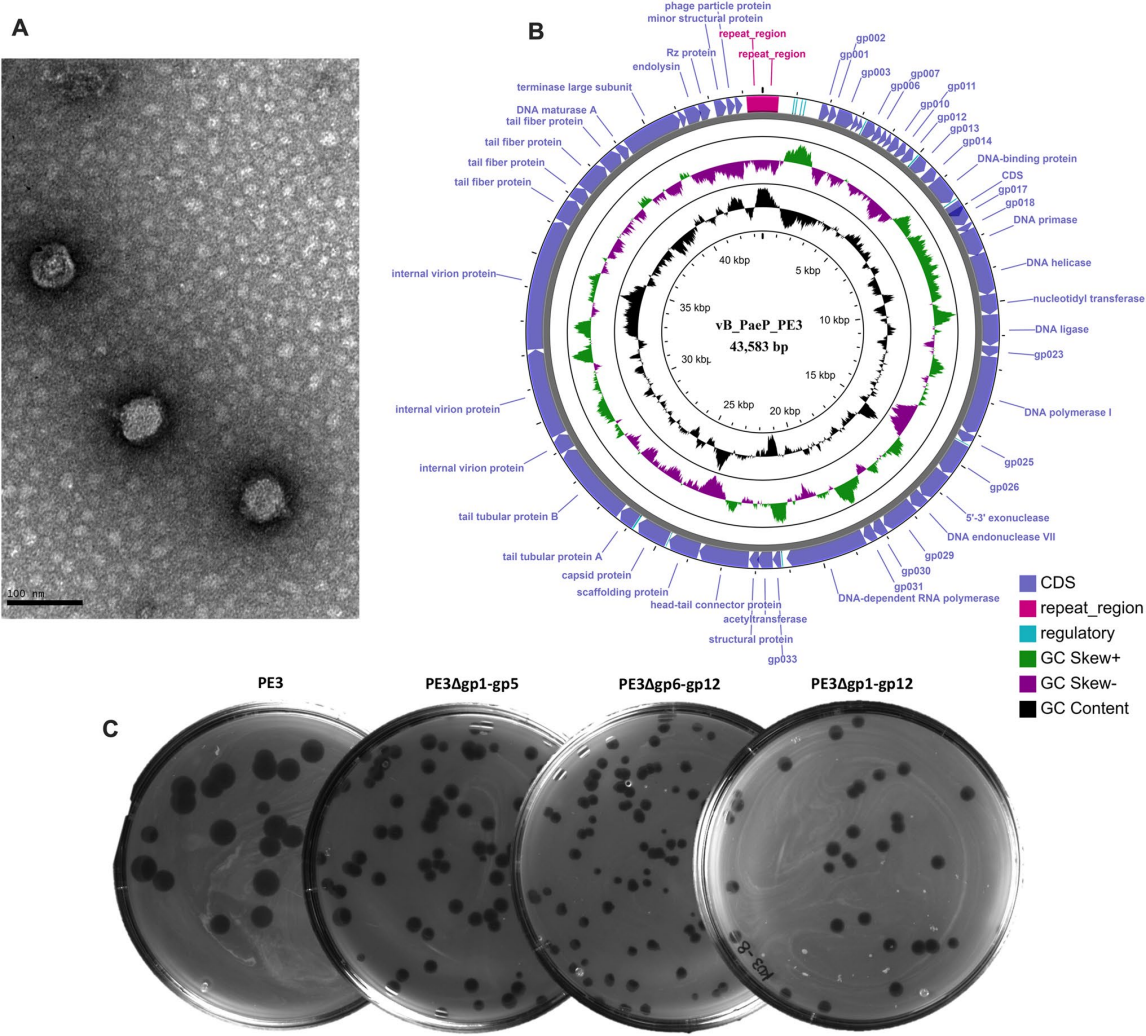
Phage characterization. (**A**) TEM observation of phage PE3. The bar indicates 100 nm. (**B**) Circular view of phage PE3 genome. Created using CGView server^16^. (**C**) Phage plaques of the wild-type and synthetic phages.

The lytic phage PE3 was isolated from a wastewater treatment plant using the *P. aeruginosa* PAO1 as host for the enrichment procedure. After isolation and propagation, the phage was tested against a panel of 28 *P. aeruginosa* clinical isolates^14^ and revealed to be quite specific, being able to infect 7 of them (Table S1). The morphological characterization by TEM (Fig. 1A) revealed that phage PE3 has a short and noncontractile tail^15^.

Phage PE3 was further characterized by whole genome sequencing. The data obtained from sequencing revealed that PE3 genome consists of a 43,583 bp double-stranded DNA with a GC content of 62.3% and two 479 bp direct terminal repeats (DTRs). Phage PE3 encodes 55 predicted CDSs with lengths ranging between 120 to 4014 bp (Fig. 1B)^16^. Based on BLASTP analysis, 30 of the predicted CDSs have assigned functions while the other 25 encode hypothetical proteins (Table S2). The genome sequencing analysis together with TEM revealed that this phage has the defining characteristics of the *Autographiviridae* family, which was until 2019 considered a subfamily of *Podoviridae*^17^. This family includes phages similar to T7 with genomes composed of linear terminally redundant dsDNA encoding a RNA polymerase^17^. The genome analysis of phage PE3 further revealed that this phage is most probably virulent as it does not encode genes known to be associated with lysogeny. In addition, no tRNAs were detected and, based on BLASTN search, we found that phage vB_PaeP_PE3 shares high homology with phages vB_PaeP_PAO1_1-15pyo^18^ and LUZ19^9^.

### Efficient assembly of synthetic phages with reduced genomes

**Figure 2:**
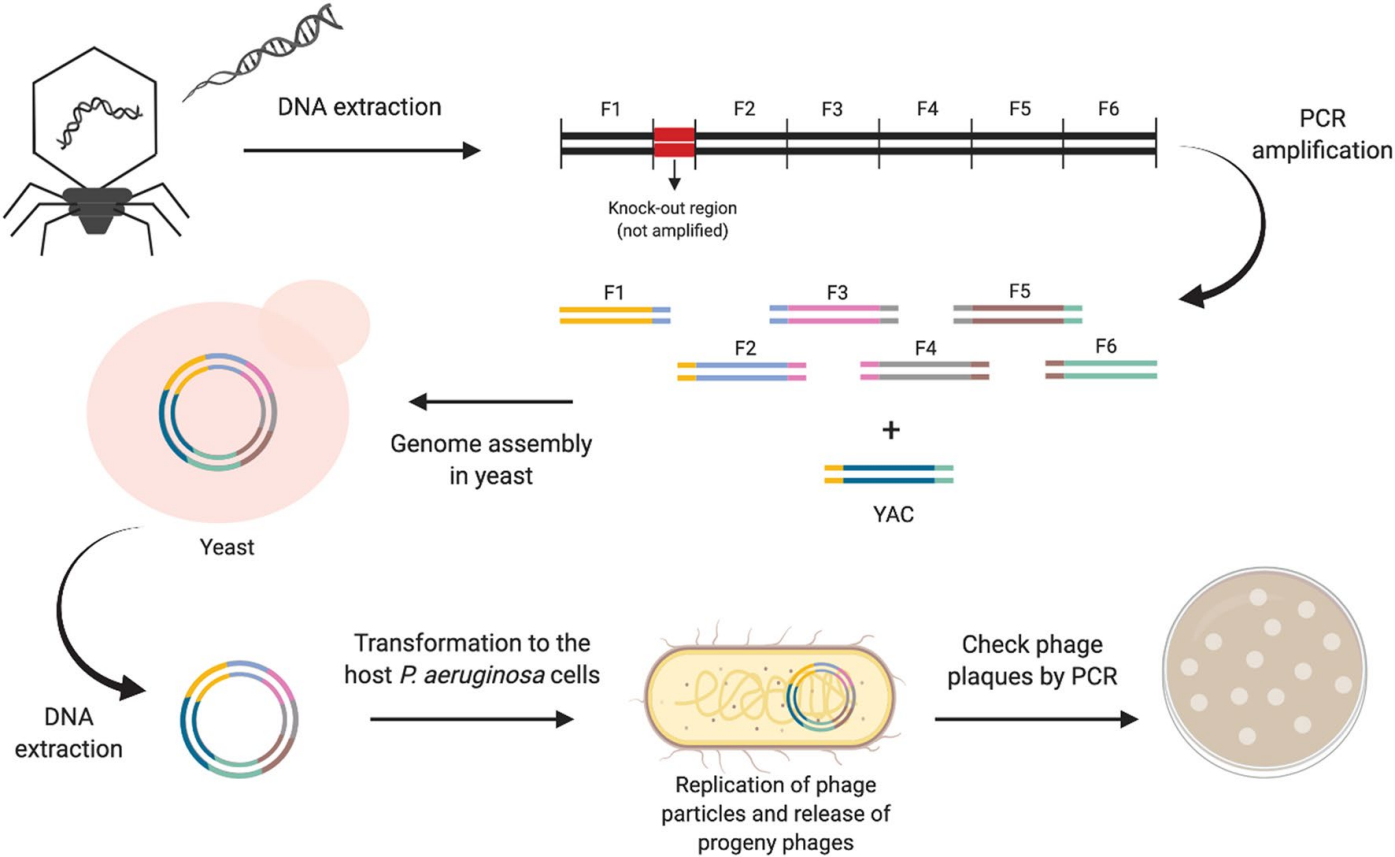
Workflow to build synthetic phages with reduced genomes. Phage DNA is used as template to generate overlapping PCR products covering the entire phage genome with the exception of the knock-out region. The PCR products are co-transformed into yeast cells along with the linearized YAC, where the phage genome is assembled. The phage genome captured into the YAC is then extracted from yeast cells and transformed to the host *P. aeruginosa* cells to generate infectious phage particles. This figure was created with BioRender.com and exported under a paid subscription.

**Figure 3:**
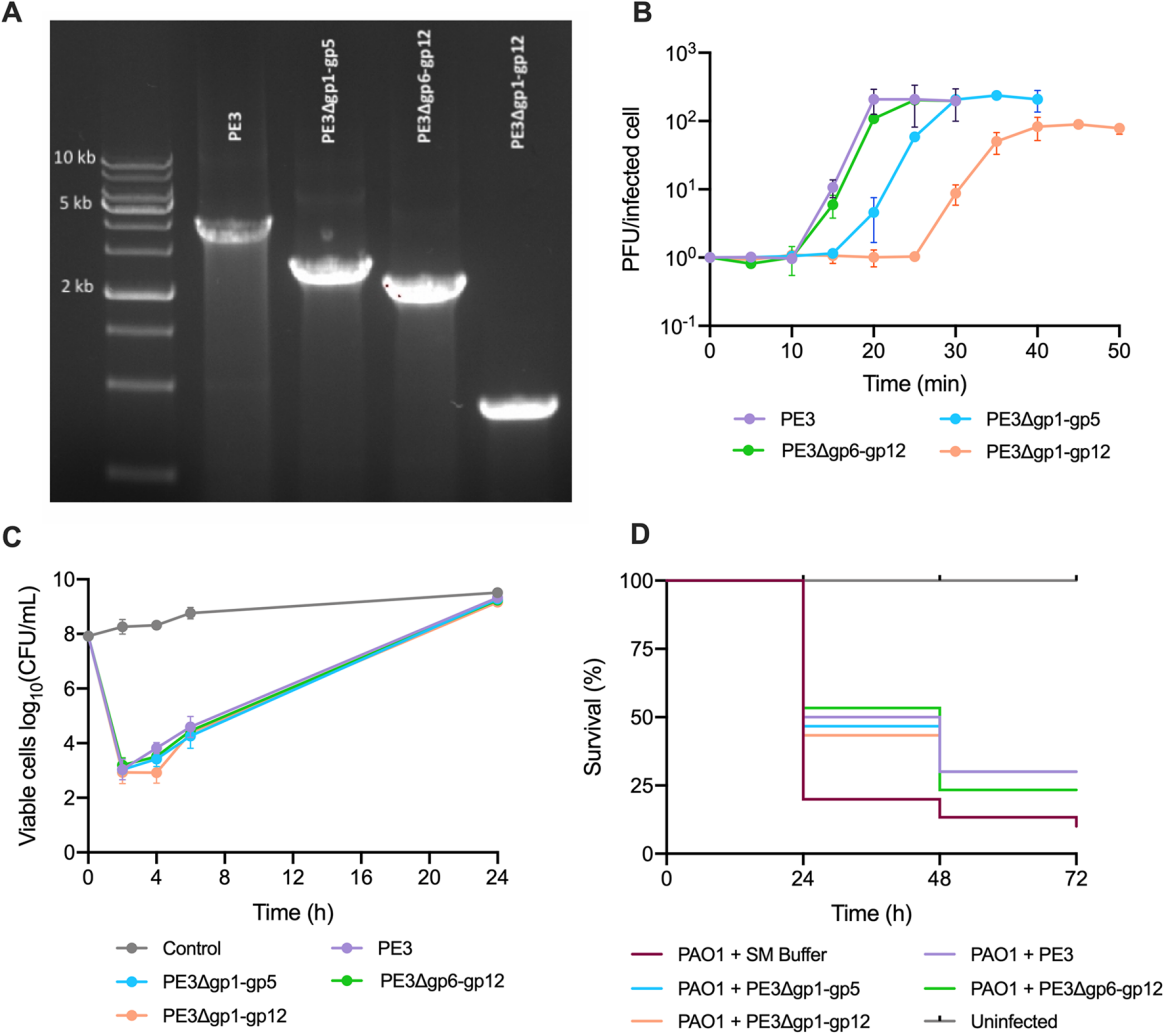
Wild-type phage versus synthetic phages. (**A**) PCR-based confirmation of the deletions in the genome of phage PE3. (**B**) One-step growth curves of wild-type (PE3) and synthetic phages phages (PE3Δgp1–gp5, PE3Δgp6–gp12 and PE3Δgp1–gp12). (**C**) Treatment of *P. aeruginosa* PAO1 log-phase cultures with the wild-type phage or synthetic phages using a MOI of ~ 5. No phage was added in the control. Error bars represent SD from three independent experiments. (**D**) *G. mellonella* larvae were injected with either PBS (uninfected group) or *P. aeruginosa* PAO1 followed by a subsequent administration of wild-type or synthetic phages at a MOI of ~ 100 or SM Buffer in the non-treated group. Survival curves represent three independent experiments, each with 10 larvae per treatment group.

A large part of the genes encoded in phage genomes do not have assigned functions, which raises the question about whether these genes may play an essential role in phage replication and bacterial killing, or if they are unnecessary and can therefore be removed from phage genomes without a detrimental effect on their antibacterial activity. Taking this into consideration, we wondered about the possibility to build synthetic phages with reduced genomes by removing genes encoding hypothetical proteins that are not essential for phage viability and replication. At the first part of the annotated sequence of phage PE3 it is possible to find two modules of genes encoding hypothetical proteins separated by regulatory elements: the first module is between gp1 and gp5 and the second between gp6 and gp12. Based on this information, we aimed to understand if by removing each of these regions of genes, the phage would still be viable. Using the yeast-based phage-engineering platform^12^, we built two fully synthetic phages, vB_PaeP_PE3Δgp1-gp5 (short name PE3Δgp1–gp5) and vB_PaeP_PE3Δgp6–gp12 (short name PE3Δgp6–gp12), each of them encoding knock-outs between gp1–gp5 and gp6–gp12, respectively. This was achieved by amplifying the whole phage genome of phage PE3, with the exception of the knock-out regions, by PCR in overlapping fragments using specific sets of primers (Table S3). The six PCR products spanning the phage genome and the linearized YAC carrying homologous “arms” with the extremities of phage genome were successfully assembled in yeast as a consequence of the gap repair system that joins each fragment to the adjacent, resulting in a full phage genome captured in the YAC (Fig. 2)^12^. The yeast transformants were then screened by yeast colony PCR to confirm the correct assembly of the phages using a set of primers located upstream and downstream the knock-out regions (Table S4), which will result in smaller PCR products comparatively to the wild-type phage. To understand if these knock-outs would affect phage’s viability, the YAC-phage DNA was extracted from yeast cells and transformed into the *P.* *aeruginosa* PAO1 host, where phage genes can be transcribed and generate functional phages. In fact, phage plaques were observed after plating. The resulting plaques were checked by PCR using the set of primers described above and revealed that the knock-outs were successfully performed on phage genome (Fig. 3A) and yielded viable phage particles, which were then propagated for further characterization. This result clearly indicates that the first 12 genes encoded in the genome of phage PE3 are non-essential for phage viability and propagation.

Since we succeeded in separately removing 5 and 7 genes from PE3 genome, we decided to design and synthesize a third synthetic phage by removing the first 12 genes encoding hypothetical proteins (gp1–gp12) corresponding to a knock-out of 3194 bp. Using the same phage-engineering strategy, the construct was assembled in yeast and the chimeric phage vB_PaeP_PE3Δgp1–gp12 (short name PE3Δgp1–gp12) was successfully obtained (Fig. 3A). Although several phage-engineering strategies have been developed and proved to be efficient in editing genomes of virulent phages^19–21^, none of them had been previously applied to engineer *P.* *aeruginosa* phages. Therefore, we opted for the yeast-based phage-engineering strategy because it was already demonstrated to be a robust platform to capture and/or manipulate genomes of phages infecting different bacterial species, including *P.* *aeruginosa*^12^.

### Synthetic phages versus wild-type phage: characterization

In order to better understand the impact of the knock-outs on phage behavior, the chimeric phages were characterized and compared to the wild-type phage. First, we noticed that the morphology of the phage plaques was slightly different from the wild-type phage, showing a reduced size (Fig. 1C). Then, we assessed the host range of the chimeric phages using the same panel of strains we used for the wild-type phage (Table S1). Surprisingly, not all phages displayed the same host range. Although PE3 and PE3Δgp6–gp12 share the same host range infecting 7 out of 28 clinical isolates, the other two chimeric phages were only able to infect 4 of the 7 strains, causing lysis from without on the others. Next, the phage growth parameters were evaluated by performing one-step-growth curves. The results revealed that the wild-type phage PE3 and the chimeric phage PE3Δgp6–gp12 have a similar growth curve, while phages PE3Δgp1–gp5 and PE3Δgp1–gp12 have 5 min and 15 min longer latent periods, respectively (Fig. 3B). Regarding the burst sizes, the values obtained for phages PE3Δgp1–gp5 (211 PFU/infected cell) and PE3Δgp6–gp12 (203 PFU/infected cell) were similar with the wild-type phage (206 PFU/infected cell). However, phage PE3Δgp1–gp12 has a significantly lower burst size of 83 PFU/infected cell.

The genomes of the three synthetic phages were sequenced to understand if mutations were introduced in the genomes due to PCR amplification. The sequencing results revealed three point mutations for all phages, comparatively with wild-type phage. To confirm these results, the three regions encoding the mutations were amplified by PCR and sequenced by Sanger. This was also performed for the wild-type phage. However, for phages PE3Δgp1–gp5 and PE3Δgp6–gp12 only two of the point mutations were confirmed by Sanger sequencing. One of these mutations is shared by both phages, as they were built at the same time and some PCR products used for yeast recombination were the same for both, and corresponds to a G → A transition on gp47, which encodes a tail fiber protein. Phage PE3Δgp1–gp5 also carries a silent mutation C → T in the endolysin gene (gp51) while phage PE3Δgp6–gp12 carries a C → A transversion resulting in a Ser to Tyr change in the tail fiber gene (gp46). The sequencing of phage PE3Δgp1–gp12 revealed a silent mutation G → A in gp37, which encodes a scaffolding protein, and two additional point mutations in genes encoding tail fiber proteins: a C → A transversion in gp46 that results in a Gln to Lys change, and a A → G transition in gp47 that results in a Asn to Asp change.

### The in vitro and in vivo antibacterial efficacy of the synthetic phages is not impaired by genome knock-outs

In order to understand if the knock-outs performed on PE3 phage genome had some impact on its antibacterial performance, in vitro and in vivo efficacy assays were performed using the wild-type and the chimeric phages.

For the in vitro experiments, phages were added to log-phase bacterial cultures at a MOI of ~ 5 and the phage infection was followed for 24 h. As observed in Fig. 3C (Table S5), all the phages revealed a similar behavior and no significant differences were found among them (*p* > 0.01). Two hours after adding phages to the cultures, the number of *P. aeruginosa* PAO1 viable cells was reduced by more than 5 orders-of-magnitude for all the phages, comparatively with the control (non-infected culture) (*p* < 0.01). After this time point, the number of viable cells started increasing and, 24 h after the beginning of phage infection, no differences were observed between treated and non-treated cultures (*p* > 0.01), indicating the fast proliferation of bacteriophage-insensitive mutants (BIMs). The emergence of BIMs within a short time after phage infection is very common for *P. aeruginosa* species and has already been widely studied^22–24^. Despite this fact, the results obtained here clearly indicate that phages’ performances were not affected by genome knock-outs as no differences were observed between the wild-type and chimeric phages.

As the in vitro assays are usually unable to mimic the complexity of the environments found in vivo, we used a *G. mellonella* infection model to compare the efficacy of the wild-type versus synthetic phages. After bacterial challenge, the larvae were injected either with phages (treated groups) or with SM Buffer (non-treated group). One control group was used by injecting larvae with PBS and SM Buffer to monitor the killing due to injection trauma (uninfected group). As shown in Fig. 3D, the survival rates of the larvae in the untreated group were 20%, 13% and 10% after 24, 48 and 72 h of bacterial infection, respectively (Table S6). The groups treated with the different phages revealed significantly higher survival rates for all time points when compared with the untreated group (*p* < 0.05). For instance, the treatment of larvae with the wild-type phage resulted in a survival rate of 50% after 24 h of infection and 30% survival after 48 and 72 h. The treatment of infected larvae with the synthetic phages resulted in survival rates similar to the wild-type phage and no significant differences were found among the treated groups. Altogether, these data evidence that the genome knock-outs do not impair phages performance both in vitro as well as in vivo. In addition, no cytotoxic effects were observed in the larvae when injected only with wild-type or chimeric phages with concentrations up to 10^8^ PFU/mL during the course of 72 h (data not shown).

## Discussion

A minimal cell is usually defined as a cell carrying only essential genes, but this definition can be controversial as the essential functions can be dependent on the environmental conditions^25,26^. Some studies have already addressed this topic for a number of bacterial species by identifying sets of essential genes^26,27^. For instance, based on transposon mutagenesis, Hutchison III et al.^25^ were able to classify bacterial genes as essential or non-essential, which enabled a rational design and posterior synthesis of a *M. mycoides* minimal genome. However, when it comes to phages, there is still a limited knowledge about which genes are essential or not, even though transcriptomics are bringing important insights in this field^9–11^.

Despite the fact that phage genomes are relatively small, phage particles are extremely abundant in the biosphere and they display a huge genetic diversity and novelty^28^. It is therefore predicted that most of the phage-encoded genes (about 80%) have unknown functions^28^, which might constitute a barrier for their therapeutic application. Taking this into consideration, in this work we intended to design and generate synthetic phages with reduced genomes in order to encode a minor number of genes with unknown functions. On the other hand, we aimed to study whether the genomic deletions made on phages influence their performance. To address this, three synthetic phages were built by removing three sets of genes encoding hypothetical proteins from the genome of the newly isolated *P. aeruginosa* phage PE3.

Phages PE3Δgp1–gp5 and PE3Δgp6–gp12 were initially designed to understand if the deletion of each module of genes would result in viable phage particles. As both constructs resulted in viable phages, this clearly revealed that all the genes comprised in the two knock-out regions of the phage genome (gp1–gp5 and gp6–gp12) are non-essential. This led us to build a third synthetic phage—PE3Δgp1–gp12—lacking both sets of genes that were shown to be unnecessary for phage viability and replication. The characterization of the chimeric phages comparatively to the wild-type phage revealed that the phage with the larger genomic deletion, PE3Δgp1–gp12, had a longer latent period and a lower burst size. However, this feature doesn’t seem to influence its performance in vitro and in vivo but elucidates that, although the set of genes from gp1 to gp12 are non-essential, some genes might have a role in the early stage of phage infection. The gene or genes that might influence phage-host interaction are probably located between gp1 and gp5 as the synthetic phage PE3Δgp1–gp5 also presented a longer latent period comparatively with the wild-type phage PE3, while phage PE3Δgp6–gp12 showed the exact same behavior as the wild-type. This similarity between phage PE3Δgp6–gp12 and the wild-type phage was also observed when analyzing their host ranges. While these two phages share the same host range, the other two phages lost the ability to infect 3 of the clinical strains being only able to cause them lysis from without, which indicates that might not be a problem of interaction with phage receptors. However, the cause for this host-range change remains to be explained. Apart from the knock-outs, the whole genome sequencing only revealed two point mutations in the genomes of phages PE3Δgp1–gp5 and PE3Δgp6–gp12 and three point mutations in the genome of phage PE3Δgp1–gp12. These mutations were probably introduced by PCR reactions during amplification of phage DNA fragments for assembly but do not seem to influence phages’ antibacterial behavior. The introduction of mutations during PCR amplification can happen, even at higher rates. For example, in a study developed by Smith et al. in which the genome of phage φX174 (5386 bp) was assembled from synthetic oligonucleotides, the synthetic phage DNA showed lower infectivity than the natural phage DNA, which was attributed to PCR-generated mutations that reached 1 per 500 bp^29^.

To further understand if the genomic knock-outs impaired the in vivo antibacterial performance of the phages, we used a *G. mellonella* infection model. The results showed that the efficacy of the chimeric phages to rescue larvae from infection was not affected by the genomic manipulations, suggesting that phage genomes can be redesigned to remove genes without relevant functions. The elimination of genes with unknown functions that proved to be unnecessary can make phages more attractive for therapeutic applications. In addition, the deletion of unnecessary genes creates some room in phage genomes, paving the way to introduce other genes of interest that can potentiate their antibacterial activity.

In conclusion, here we demonstrated that although phage genomes are usually very compact and well organized, they carry many non-essential genes that can be removed from its genome without a detrimental effect to the infection. In this work we were able to remove 48% of the genes encoding hypothetical proteins, building phages in which the vast majority of the genes have assigned functions. In this way, we proved that 12 of the 55 genes encoded in the genome of PE3 phage are non-essential. Although the yeast-based phage-engineering platform used in this work was shown to be a reliable and efficient strategy for genome editing *P. aeruginosa* phages, it might be limited to bacterial hosts amenable to genetic manipulation.

Despite the major developments in phage-engineering tools and platforms and the increasing interest in this field^5,6^, there is still a limited number of reports on phage engineering, which target a narrow range of bacterial hosts. However, we envisioned that in a near future, the new advances in synthetic biology field will enable to quickly design and create specialized phages “a la carte” to a wide range of bacterial species. Finally, the in vivo experiments showed that engineered phages can be safely used and might constitute an interesting approach to treat *P. aeruginosa* infections in the future.

## Materials and methods

### Strains, plasmids and primers

Phage vB_PaeP_PE3 (short name PE3, see Table S7) was isolated in this study. The three synthetic phages PE3Δgp1–gp5, PE3Δgp6–gp12 and PE3Δgp1–gp12 are listed in Table S7. The host bacterial strain of PE3 phage is the reference strain *P. aeruginosa* PAO1 (DSM22644), obtained from the German Collection of Microorganisms and Cell Cultures (DSMZ-Deutsche Sammlung von Mikro-organismen und Zellkulturen.) The *P.* *aeruginosa* clinical strains used to evaluate the host range of phages belong to the lab collection^14^. The *Saccharomyces cerevisiae* BY4741 (*MAT*a *his3*Δ*1* *leu2*Δ*0* *met15*Δ*0* *ura3*Δ*0*) and the yeast centromere vector pRS415 (ATCC 87520) with *LEU2* marker were obtained from laboratory stocks. All primers used in this study are listed in Table S4.

### Culture conditions

*Pseudomonas* *aeruginosa* PAO1 was grown at 37 °C in lysogeny broth (LB) or LB agar (LB with 1.2% (w/v) of agar). *S. cerevisiae* BY4741 was cultured in YPD (1% (w/v) Bacto Yeast Extract, 2% (w/v) Bacto Peptone and 2% dextrose (w/v)) or YPD agar at 30 °C.

### Phage isolation

The bacteriophage PE3 used in this study was isolated from a wastewater treatment plant (Braga, Portugal) using *P. aeruginosa* PAO1 as the host strain for the enrichment procedure and following the protocol described by Azeredo et al.^30^.

### Phage propagation and titration

Phage production and titration were performed using the double agar overlay technique as described previously^22,30^.

### Transmission electron microscopy

Phage PE3 was morphologically characterized by Transmission Electron Microscopy (TEM) using a procedure previously described^31^. Briefly, phage particles were collected by centrifugation (25,000×*g*, 4 °C, 60 min). The sedimented particles were washed twice with tap water and centrifuged again. Phages were then deposited on copper grids with carbon-coated Formvar films, stained with 2% uranyl acetate (pH 4.0) and examined using a Jeol JEM 1400 transmission electron microscope (Tokyo, Japan).

### Phage DNA isolation

The isolation of phages’ DNA was performed using an adapted version of the protocol described by Ando et al.^12^. 150 mL of phage lysates were centrifuged (9000×*g*, 4 °C, 15 min) and filtered (0.22 μm) to remove cellular debris. Purified lysates were then mixed with 200 μL of buffer L1 [20 mg/mL RNase A, 6 mg/mL DNase I, 0.2 mg/mL BSA, 10 mM EDTA, 100 mM Tris-HCl, 300 mM NaCl, pH 7.5] for 30 min at 37 °C. After that, 30 mL of cold buffer L2 [30% (w/v) polyethylene glycol (PEG) 6000, 3 M NaCl] were added and incubated on ice under agitation (90 rpm) for 1 h. The suspension was then centrifuged (9000×*g*, 4 °C, 30 min), the supernatant discarded, and the pellets resuspended in a total of 9 mL of buffer L3 (100 MM Tris-HCl, 100 mM NaCl, 25 mM EDTA, pH 7.5). Nine mL of buffer L4 [4% (w/v) SDS] were added, mixed gently and the tubes were incubated at 70 °C for 20 min and then allowed to cool on ice. Afterwards, 9 mL of buffer L5 (2.55 M potassium acetate, pH 4.8) were added and mixed gently by inverting the tubes. This solution was centrifuged (10,000×*g*, 4 °C, 1 h) and the supernatant was saved and passed through a Qiagen-tip 100 column according to the manufacturer’s instructions. The eluted DNA was then precipitated by adding 0.7 volumes of isopropanol and centrifuged (10,000×*g*, 4 °C, 30 min). The pellet was sequentially washed with 70% (v/v) ethanol and 95% (v/v) ethanol. After completely air-dried, the pellet was resuspended in sterile water and stored at − 20 °C.

### Genome sequencing

The Illumina Nextera XT library preparation kit was used for the library construction of the phage DNA samples. The generated DNA fragments (DNA libraries) were sequenced in the Illumina MiSeq platform using 300 bp paired-end sequencing reads. The raw sequence data were then automatically trimmed and the demultiplexed reads were assembled into a single contig using Geneious R11.

### In silico analysis of phage genome

The potential coding sequences (CDSs) of phages were firstly annotated using myRAST^32^. Sequence similarity searches were then manually performed with the translation of each predicted CDS against the National Center for Biotechnology Information (NCBI) protein database, using BLASTP^33^, in order to assign putative functions. Promoters were predicted using MEME^34^ and phiSITE^35^, while predicted terminators were found using ARNold^36^. The tRNAscan-SE tool^37^ was used to search for tRNAs.

### Preparation of PCR products for assembling phage genomes

All PCR products were prepared using specific sets of primers (see Table S3 and S4) and the Phusion High-Fidelity DNA Polymerase (Thermo Scientific). All the PCR fragments were excised from an agarose gel after electrophoresis and recovered using the Zymoclean Gel DNA Recovery kit (Zymo Research). Homologous overhangs to the 5′ and 3′ ends of phage genome were added to the primers used to amplify the yeast artificial chromosome (YAC) pRS415, in order to enable the subsequent capture of phage genomes into the YAC. Seven PCR products (including the YAC) were used for each yeast transformation (Table S3).

### Preparation of yeast competent cells

The preparation of yeast competent cells was performed as described by Ando et al.^12^ with minor modifications. Briefly, *S. cerevisiae* BY4741 was grown in 10 mL YPD at 30 °C for approximately 24 h. Five mL of this culture were transferred into 50 mL of YPD and incubated at 30 °C for 5 h. Cells were then harvested by centrifugation (5000×*g*, RT, 5 min), washed twice with 25 mL of water and resuspended in 1 mL of water. The cellular suspension was centrifuged again (13,000×*g*, RT, 30 s) and resuspended in 1 mL of water. One hundred microliters of this cellular suspension were used for each transformation.

### Yeast transformation

Yeast transformation was performed according to a previously described protocol with minor modifications^12^. All PCR products (phage DNA fragments and the linearized pRS415) were combined in a tube (1 μg of each DNA fragment and 200 ng linearized pRS415 in 35 μl water), and mixed with the transformation mixture [100 μL yeast competent cells, 240 μL 50% (w/v) PEG 3350, 36 μL 1 M LiAc, 50 μL 2 mg/mL salmon sperm DNA]. The mixture was incubated at 42 °C for 45 min, then centrifuged at 13,000×*g* for 30 s and resuspended in 200 μL of YPD. Transformants were selected on a complete synthetic defined medium with leucine dropout (SD-Leu) [0.67% Yeast Nitrogen Base (YNB), 0.069% CSM-Leu, 2% dextrose] agar plates at 30 °C for 3 days.

### Yeast DNA extraction of captured phage genomes

YAC-Phage DNA was extracted from yeast cells as previously described by Ando et al.^12^.

### Preparation of *P. aeruginosa* electrocompetent cells and bacterial transformation

*Pseudomonas aeruginosa* PAO1 electrocompetent cells were prepared according to a protocol described elsewhere^38^. Briefly, 6 mL of an overnight grown culture were distributed by 4 microcentrifuge tubes and the cells were harvested by centrifugation (16,000×*g*, RT, 1 min). Afterwards, each pellet was washed twice with 1 mL of 300 mM sucrose. Then, the 4 bacterial pellets were resuspended and combined in a total of 100 μL of 300 mM sucrose for each transformation.

To transform *P. aeruginosa* PAO1 cells, 100 μL of electrocompetent cells were mixed with extracted DNA (YAC-phage DNA) and the mixture was transferred to a 2 mm gap electroporation cuvette. Afterwards, a pulse was applied (25 μF, 200 Ω, 2.5 kV) and 900 μL of SOC medium were added. The cellular suspension was transferred to a tube and incubated at 37 °C for 2–3 h with agitation (200 rpm) before plaque formation assays.

### Plaque formation assays

To recover the synthetic phages, approximately 200–500 μL of the cellular suspension resulting from YAC-phage DNA electroporation were mixed with 3 mL of LB soft agar into an agar plate. After overnight incubation at 37 °C, the plates were analyzed to check for the presence of phage plaques. The resulting phage plaques were checked by PCR to confirm the correct knock-outs on the synthetic phages.

### Phage host range

The host-range of the wild-type and synthetic phages was assessed using 28 *P. aeruginosa* clinical strains^14^. One drop of phage suspensions was spotted on the lawns of the different bacterial isolates. The plates were then incubated overnight at 37 °C and the susceptibility of each host to the phage was evaluated. In the cases where lysis was observed, serial dilutions of phage suspensions were made and spotted again on the bacterial lawns to identify possible cases of lysis from without.

### Phage growth parameters

To determine phage growth parameters, one-step growth curves were performed. Briefly, 10 mL of a mid-exponential phase culture with an OD_600_ of 0.3 were centrifuged (7000×*g*, 4 °C, 5 min) and resuspended in fresh medium. Then, the phage solution was added to obtain a MOI of 0.01 and phages were allowed to adsorb for 5 min at 37 °C under agitation (120 rpm). After that, the suspension was centrifuged again (7000×*g*, 4 °C, 5 min), the pellet was resuspended in 10 mL of fresh medium and incubated at 37 °C with agitation. One sample was immediately taken before incubation and after that, samples were taken every 5 min during the course of the experiment. Each sample was serially diluted to count the plaque formation units (PFUs).

### Phage infection of suspended cultures

A *P. aeruginosa* PAO1 culture grown for 16 h was diluted 1:100 in a final volume of 20 mL and incubated at 37 °C and 120 rpm until reaching an OD_600nm_ of 0.25. At this point, the phages were added at an MOI of 5, except for the control. The suspensions were then incubated at 37 °C with agitation (120 rpm) and CFU counts were determined after 2, 4, 6 and 24 h, as described by Pires et al.^22^.

### *Galleria mellonella* infection model

Wax moth larvae *G. mellonella* were reared at 25 °C in the darkness, from egg to last instar larvae on natural diet (beeswax and pollen grains). Worms of the final instar larval stage, weighing 250 ± 25 mg, were selected to be used in the experiments. The *G*. *mellonella* survival experiment was adapted from previous studies with small changes^39,40^. Briefly, *P*. *aeruginosa* PAO1 overnight cultures were grown in LB at 37 °C with shaking. The bacterial culture was diluted in PBS to 8–9 CFU per volume of injection (5 μL). Ten larvae per group were randomly selected based on size. Using a hypodermic microsyringe, the larvae were injected with 8–9 CFU suspensions via the last left side proleg, previously surface-sanitized with alcohol at 70% (v/v). Approximately 30 min after bacterial injection, SM Buffer (5.8 g/L NaCl, 2 g/L MgSO_4_·7H_2_O, 50 mL/L 1 M Tris-HCl pH = 7.5) or phage treatment at a MOI of 100 was administered in the penultimate right proleg. In the control group, the larvae were injected with PBS and SM Buffer to monitor the killing due to injection trauma. Cytotoxicity assays were also performed by injecting larvae only with phages with titers ranging between 10^2^ and 10^6^ PFU per injection. Larvae were then incubated in petri dishes and maintained in the dark at 37 °C. The survival was monitored every 24 h for 72 h and the larvae were considered dead when there was a lack of mobility in response to touch. Kaplan–Meier survival curves were generated and analyzed with the log-rank test using GraphPad Prism.

### GenBank submission

The complete genome of the *P. aeruginosa* phage PE3 has been deposited in GenBank under the accession number MN901924.

### Statistical analysis

All data of the experiments were analyzed using GraphPad Prism version 8. The data are presented as the mean of three independent experiments and the error bars represent the SD.

## Supplementary Information

Supplementary information.

Supplementary tables.
