# Supplementary material for: Designing P. aeruginosa synthetic phages with reduced genomes
Source: Sci Rep. 2021 Jan 25;11:2164. doi: 10.1038/s41598-021-81580-2 (PMC7835345; doi:10.1038/s41598-021-81580-2)

**Supplementary information**

**Designing *P. aeruginosa* synthetic phages with reduced genomes**

Diana P. Pires^1*^, Rodrigo Monteiro^1^, Dalila Mil-Homens^2^, Arsénio Fialho^2,3^, Timothy K. Lu^4^, Joana Azeredo^1*^

^1^ CEB - Centre of Biological Engineering, Universidade do Minho, Campus de Gualtar, Braga, Portugal

^2^ Institute for Bioengineering and Biosciences (iBB), Instituto Superior Técnico, Lisboa, Portugal

^3^Department of Bioengineering, Instituto Superior Técnico, Universidade de Lisboa, Lisboa, Portugal

^4^ Department of Electrical Engineering and Computer Science and Department of Biological Engineering, Synthetic Biology Center, Massachusetts Institute of Technology, 77 Massachusetts Avenue, Cambridge, MA 02139, USA

*Corresponding authors:

Diana P. Pires – [priscilapires@deb.uminho.pt](mailto:priscilapires@deb.uminho.pt)

Joana Azeredo – [jazeredo@deb.uminho.pt](mailto:jazeredo@deb.uminho.pt)

**Table S1**. Lytic spectra of phages

| **Strain** | **PE3** | **PE3Δgp1-gp5** | **PE3Δgp6-gp12** | **PE3Δgp1-gp12** |
| --- | --- | --- | --- | --- |
| PAO1 | + | + | + | + |
| 1 | - | - | - | - |
| 2 | - | - | - | - |
| 3 | - | - | - | - |
| 4 | - | - | - | - |
| 5 | + | + | + | + |
| 6 | + | + | + | + |
| 7 | - | - | - | - |
| 8 | - | - | - | - |
| 9 | - | - | - | - |
| 10 | - | - | - | - |
| 11 | - | - | - | - |
| 12 | + | LFW | + | LFW |
| 14 | - | - | - | - |
| 15 | - | - | - | - |
| 16 | - | - | - | - |
| 17 | + | LFW | + | LFW |
| 18 | - | - | - | - |
| 19 | - | - | - | - |
| 20 | - | - | - | - |
| 21 | - | - | - | - |
| 22 | - | - | - | - |
| 23 | + | + | + | + |
| 24 | - | - | - | - |
| 25 | - | - | - | - |
| 26 | H | H | H | H |
| 27 | H | LFW | H | LFW |
| 28 | - | - | - | - |
| 29 | - | - | - | - |

+ lysis; - no lysis; H - hazy halo; LFW - Lysis from without

**Table S5**. Detailed data from Figure 3C

| **Time after treatment** | **Viable cells, log_10_ (mean ± SD CFU/mL)** | | | | |
| --- | --- | --- | --- | --- | --- |
|  | **Control**  **(no phage)** | **PE3** | **PE3Δgp1-gp5** | **PE3Δgp6-gp12** | **PE3Δgp1-gp12** |
| 0h | 7.917 ± 0.147 | 7.917 ± 0.147 | 7.917 ± 0.147 | 7.917 ± 0.147 | 7.917 ± 0.147 |
| 2h | 8.262 ± 0.271 | 3.040 ± 0.379 | 3.033 ± 0.184 | 3.198 ± 0.271 | 2.933 ± 0.413 |
| 4h | 8.317 ± 0.084 | 3.817 ± 0.206 | 3.418 ± 0.274 | 3.509 ± 0.239 | 2.922 ± 0.387 |
| 6h | 8.761 ± 0.213 | 4.592 ± 0.384 | 4.261 ± 0.455 | 4.456 ± 0.240 | 4.388 ± 0.238 |
| 24h | 9.512 ± 0.181 | 9.332 ± 0.034 | 9.278 ± 0.127 | 9.239 ± 0.105 | 9.159 ± 0.106 |

**Table S6**. Detailed data from Figure 3D

| **Treatment** | **% Survival** | | |
| --- | --- | --- | --- |
|  | **24h** | **48h** | **72h** |
| PBS + SM Buffer (uninfected) | 100 | 100 | 100 |
| PAO1 + SM Buffer | 20 | 13 | 10 |
| PAO1 + PE3 | 50 | 30 | 30 |
| PAO1 + PE3Δgp1-gp5 | 47 | 30 | 30 |
| PAO1 + PE3Δgp6-gp12 | 53 | 23 | 23 |
| PAO1 + PE3Δgp1-gp12 | 43 | 30 | 30 |

**Table S7**. Phages used in this study

| Phage (Original Name) | Phage (Short Name) | Source | Description |
| --- | --- | --- | --- |
| vB_PaeP_PE3 | PE3 | This study | Wild-type phage |
| vB_PaeP_PE3Δgp1-gp5 | PE3Δgp1-gp5 | This study | vB_PaeP_PE3 lacking genes gp1-gp5 |
| vB_PaeP_PE3Δgp6-gp12 | PE3Δgp6-gp12 | This study | vB_PaeP_PE3 lacking genes gp6-gp12 |
| vB_PaeP_PE3Δgp1-gp12 | PE3Δgp1-gp12 | This study | vB_PaeP_PE3 lacking genes gp1-gp12 |

**Figure S1**. Full length gel from Figure 3A


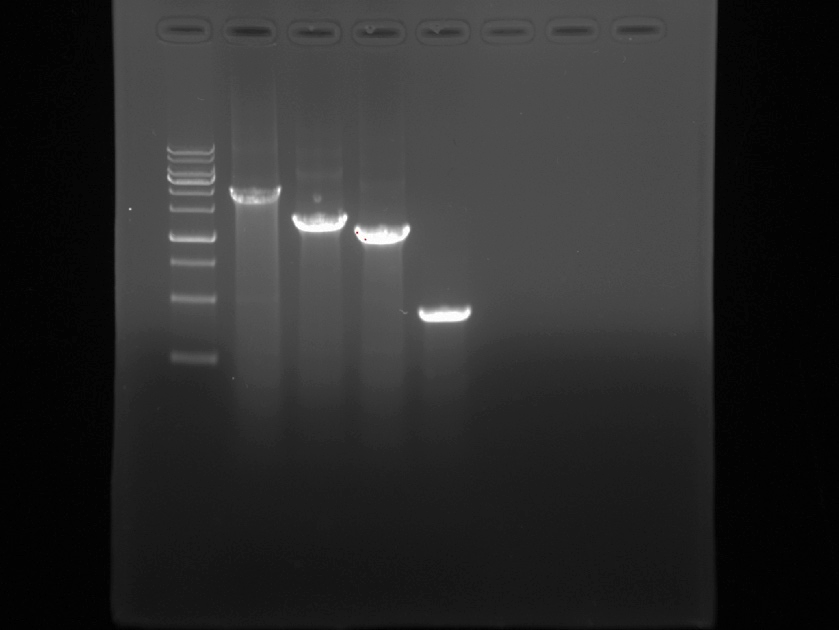

Supplement: Supplementary file 1 — Supplementary information. [file 41598_2021_81580_MOESM1_ESM.docx]
